# *Achyranthes aspera* Root Extracts Induce Human Colon Cancer Cell (COLO-205) Death by Triggering the Mitochondrial Apoptosis Pathway and S Phase Cell Cycle Arrest

**DOI:** 10.1155/2014/129697

**Published:** 2014-10-27

**Authors:** Shagun Arora, Simran Tandon

**Affiliations:** Department of Biotechnology and Bioinformatics, Jaypee University of Information Technology, Waknaghat, Solan, Himachal Pradesh 173234, India

## Abstract

*Achyranthes aspera* (AA) has been used traditionally for the cure of various disorders. However, the action of root extracts of AA as anticancer agent and its cellular mechanism remain unclear. The aim was to screen the antitumor effect of ethanolic (EAA) and aqueous (AAA) root extracts on the growth of colon cancer COLO-205 cells by testing their cytotoxicity, followed by their effect on clonogenicity, migration, and induction of apoptosis. Mechanisms leading to apoptosis and cell cycle arrest were also investigated by expression studies of caspase-9, caspase-3, Bax, Bcl-2, p16, p21, and p27 genes, followed by flow cytometric analysis for cell cycle distribution. Cytotoxicity screening of AA extracts indicated greater cytotoxic activity of AAA extract against COLO-205 cells. A series of events marked by apoptosis revealed loss of cell viability, chromatin condensation, and DNA fragmentation in AAA treated cells to a greater extent. The mRNA expression levels of caspase-9, caspase-3, Bax, p16, p21, and p27 were markedly increased in the AAA treated cells, along with decreased Bcl-2 expression. The cell cycle arrest at S phase was detected by flow cytometric analysis after treatment with AAA. Overall the study signifies the aqueous extracts as a promising therapeutic candidate against cancer.

## 1. Introduction

Despite significant advances toward targeted therapy and screening techniques, colon cancer continues to be a chronic disease worldwide, being the third leading cause of death in men and the second in women globally. According to the Globocan 2012 Cancer Fact Sheet, about 1.36 million new cases of colon cancer were clinically diagnosed, with number of deaths being 0.69 million [1]. In the development of cancer, evasion of apoptosis is one of the major factors resulting in overpopulation of cancer cells. Apoptosis is an active form of cell death guided by a set of prosurvival and antisurvival genes [2]. There is a strong corelation between loss of apoptotic control and cancer initiation and progression, as tumor cells lose their ability to activate the death signalling pathway [3]. Other than apoptosis, deregulated cell-cycle control is a key feature of cancer progression. In normal cells, the cell cycle begins or stops only in response to proliferation-enhancing or retarding signals, respectively, which however is not seen in cancer cells. As a result of this, their proliferation remains unchecked [4].

Although conventional chemotherapeutic drugs induce cell death, they are limited by their toxicity to normal cells. Identification of natural agents in form of either plant extracts or a bioactive compound, which successfully exhibits apoptotic and cell cycle modulating properties and at the same time shows limited toxicity to normal cells, is therefore essential [5].

Any health care practices, which do not form a part of conventional western medicine, are referred to as complementary and alternate therapies (CAM). According to WHO, 80% of the world's population relies upon the use of traditional herbal medicines for general wellness [6]. An effective strategy for identifying potential anticancer molecules should be based upon validation of those plants whose ethnobotanical and ethnopharmacological use have shown promise rather than mass screening of plants in general. The use of herbs, plants, and homeopathic, Ayurvedic, and traditional medicines has been outlined as a part of CAM therapies from ancient times; however the effectiveness of such therapies against cancer management and prevention is still uncertain due to either lack of scientific data or safety related issues. An understanding of the use of CAM therapies in mainstream cancer treatment therefore is the need of the hour.* Achyranthes aspera* (AA) is a known traditional herb, which belongs to family Amaranthaceae. All parts of AA are used in traditional system of medicines such as seeds, roots, and shoots. AA is used for the management of various diseases such as malaria, dysentery, sinuses, asthma, piles, night blindness, hypertension, and diabetes [7]. The leaf extracts of AA have shown antioxidant, diuretic, antidepressant, hepatoprotective, wound-healing, and cancer chemopreventive effects [8–11]. Other than leaves, roots of AA possess anti-inflammatory and immunomodulatory effects [12, 13]. Although the use of AA which started in the Vedic period continues to be a part of present era, the experimental studies into the effective role of roots of* Achyranthes aspera* against colon cancer management and its mechanism of action are still limited.

Therefore, the aims of this study were the following: (1) to evaluate the cytotoxic activities of the AA root extracts against COLO-205 cells and (2) to further investigate the molecular mechanism of apoptosis triggered by the best extract.

## 2. Materials and Methods

### 2.1. Sample Collection

The dried roots of AA were procured from Natural Remedies Pvt. Ltd. at Bangalore, India. The voucher specimen is available at Natural Remedies.

### 2.2. Preparation of Extracts

#### 2.2.1. Preparation of Ethanolic Extract of* Achyranthes aspera* Roots (EAA)

10 grams of dried roots was ground to a powder and suspended in 100 mL of 70% ethanol at room temperature overnight to prepare 10% (w/v) extract. The supernatant was filtered by Whatman filter number 2. The filtrate obtained was further concentrated in rotary evaporator (at 50°–60°C) under reduced pressure leaving a dark brown residue. The EAA thus obtained was transferred to a Petri dish and kept in an oven (50°C) until the solvent was completely evaporated. The average yield was 8%. It was stored in air tight container at −20°C till further use.

#### 2.2.2. Preparation of Aqueous Extract of* Achyranthes aspera* Roots (AAA)

10 grams of dried roots which had been ground to a powder was suspended in 100 mL of water at room temperature overnight to prepare 10% (w/v) extract. The supernatant was filtered by Whatman filter number 2. The filtrate was then lyophilized and the average yield was 12%. The powder was stored at −20°C till further use.

For the cell culture based assays, 100 mg/mL stock solutions were prepared by dissolving extracts in DMSO and filtered through 0.22 micron filters and stored at −20°C till further use. The stocks were diluted in cell culture media at the appropriate concentrations, prior to addition to cells.

### 2.3. Cell Culture

COLO-205 cells were obtained from NCCS, Pune, India. The cells were cultured in RPMI media supplemented with 1% antibiotic (penicillin-streptomycin), 10% FBS, and 1% sodium pyruvate. All the chemicals used for cell culture were obtained from Sigma-Aldrich, India. The cells were maintained at 37°C in a 5% CO_2_ incubator.

### 2.4. Determination of Total Phenolic Content

The total phenolic content (TPC) was determined using Folin-Ciocalteu reagent. To 50 *µ*L of AA root extracts, 3.5 mL of water and 250 *µ*L FC (2 N Folin-Ciocalteu) reagent were added. The solution was mixed and incubated for 8 minutes at room temperature. To this, 750 *µ*L of 20% sodium carbonate solution was added and incubated for another 2 hrs at room temperature. At the end of incubation, the absorbance was taken at 765 nm. The TPC value was expressed as *µ*g gallic acid equivalents (GAE)/gram of dry extract [14].

### 2.5. Determination of Antioxidant Activity

#### 2.5.1. DPPH Assay

Free radical scavenging activity was calculated using DPPH (1, 1 diphenyl-2-picrylhydrazyl) assay [14]. To 1.5 mL of AA root extracts of varying concentrations, 1.5 ml of 0.135 mM DPPH solution was added and incubated in the dark at room temperature for 30 minutes. The absorbance of the mixture was measured at 517 nm. The IC_50_ values were obtained and referred to as the concentration (*μ*g/mL) of extract that scavenges the DPPH radicals by 50%.

The calculations were as follows:

(1) DPPH  radical  scavenging  activity  (%)=[Acontrol−AtestAcontrol]×100,

where *A*_control_: absorbance of DPPH radical + methanol and *A*_test_: absorbance of DPPH radical + sample extract.

#### 2.5.2. FRAP Assay

The antioxidant activity was also evaluated by estimating the FRAP (ferric reducing ability of plasma) value. To 150 *μ*L of AA root extracts, 2850 *μ*L of FRAP solution was added and incubated for 30 minutes in the dark. Readings of the coloured product (ferrous tripyridyltriazine complex) were taken at 593 nm. Higher FRAP value indicates higher antioxidant potential. The results were expressed in *µ*M Fe (II)/gram of dry extract [14].

### 2.6. Trypan Blue Viability Assay

The dye exclusion assay was used to determine the number of viable cells present in a cell suspension. 10^5^ cells/mL was seeded in a 12-well plate and treated with different concentration of EAA and AAA (50–200 *µ*g/mL) for either 48 or 72 hours. After treatment, the cells were trypsinized and counted by a haemocytometer [15]. Cell viability was determined by following formula:

(2) $$\begin{array}{c} \%\;\text{Viability}=\frac{\text{Number}\;\text{of}\;\text{viable}\;\text{cells}\;\text{counted}}{\text{Total}\;\text{number}\;\text{of}\;\text{cells}\;\text{counted}}\times 100. \end{array}$$

### 2.7. Cytotoxicity Assay by MTT Assay

The cytotoxic activity was measured using MTT (3-(4, 5 dimethylthiazol-2-yl)-2, 5-diphenyl tetrazolium bromide) assay [15]. This colorimetric assay measures the reduction of yellow MTT to an insoluble, coloured (dark purple) formazan product by mitochondrial succinate dehydrogenase. Briefly, 10^4^ cells/well were seeded in 96-well microplates. Cells were treated with various concentrations of EAA and AAA (50–200 *µ*g/mL) for 72 hours. 5-FU (1–5 *µ*g/mL) was used as the positive control. At the end of treatment, 15 *µ*L of MTT (5 mg/mL) was added to each well and incubated for another 4 hours. The formazan products were solubilized by adding 130 *µ*L of DMSO. The optical density was measured at 570 nm using a microplate reader. The percent cell cytotoxicity was calculated by means of the following formula:

(3) $$\begin{array}{c} \%\;\text{Cell}\;\text{cytotoxicity}=100-\left\{\frac{A_{\text{control}}-A_{\text{test}}}{A_{\text{control}}}\times 100\right\}. \end{array}$$

The IC_50_ values for both EAA and AAA were determined by plotting a dose response curve and from the generated straight line equation the concentrations which lead to 50% inhibition were obtained.

### 2.8. Assessment of Growth Kinetics

To evaluate whether the extracts had any growth retarding effects, the change in growth kinetics was determined by treating cells with IC_50_ of EAA (184.1 ± 1.8 *µ*g/mL) and AAA (165.7 ± 0.6 *µ*g/mL) for 72 hours. Later the change in cell growth was observed under phase contrast microscope at 100x magnification using Nikon eclipse Ti microscope [16].

### 2.9. *In Vitro* Wound Scratch Assay

To confirm the migration capability of the COLO-205-treated cells, the scratch motility assay was carried out [17]. 10^5^ cells/well were seeded into a 12-well plate and were allowed to grow overnight to reach confluency. The monolayer was then scratched using a 20–200 *µ*L micropipette tip, washed with PBS twice to remove detached cells, and photographed at 0 hours. The cells were then treated with IC_50_ values of EAA (184.1 ± 1.8 *µ*g/mL) and AAA (165.7 ± 0.6 *µ*g/mL) for 24 hours. After incubation period of 24 hours, the cells which migrated into the scratched area were photographed under a phase-contrast inverted microscope at a magnification of 40x (Nikon eclipse Ti).

### 2.10. Clonogenic Survival Assay

Clonogenic survival assay was performed to investigate the antiproliferation capabilities of AA extracts [18]. Briefly, 5000 cells/mL were seeded in a 6-well plate and after achieving 80% confluence the cells were treated with EAA (184.1 ± 1.8 *µ*g/mL) and AAA (165.7 ± 0.6 *µ*g/mL) at their IC_50_ for 72 hours. The old media were removed and replaced with fresh complete medium and kept for another 10 days. After 14 days of incubation, the colonies were fixed with 4% paraformaldehyde at room temperature for 20 minutes and stained with 0.1% crystal violet for 10 minutes and, finally, positive colony formation (<50 cells/colony) was counted using a colony counter (DECIBEL, Digital Technologies, magnification ×1.74) and percent colony formation was calculated.

### 2.11. Antiproliferation Assay

**Figure 1:**
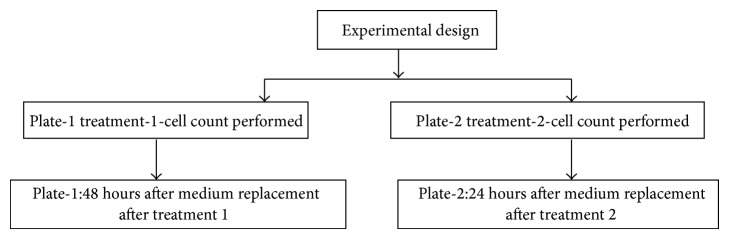
The work plan for carrying out antiproliferative assay of EAA and AAA treated cells on COLO-205 cells.

To compare the rate of cell proliferation in presence or absence of AA root extracts after specific treatment, we modified the cell viability method [15]. 0.5 × 10^5^ cells/mL were seeded in a 24-well plate and treated with different concentration of EAA and AAA (50–200 *μ*g/mL) for 48 and 72 hours. At the end of treatment, one set of cells was assayed for cell viability using trypan blue. In other sets, the old medium containing the AA extracts was replaced with fresh medium (minus extract) and incubated for a specified period, prior to growth assay. On completion of treatment, the cell count was performed. A schematic representation of the assay is depicted in Figure 1. The rationale for performing this assay was to ascertain whether the growth retarding effects were reversible, in which case the cell count would increase, or irreversible, wherein the cell count would decrease as the extracts would have had affected the cells in a permanent manner.

### 2.12. Induction of Apoptosis

#### 2.12.1. Hoechst Staining

A cell undergoing apoptosis displays the signs of nuclear condensation and DNA fragmentation which can be visualised by staining with Hoechst 33258 dye. In a 12-well plate, the cells (10^5^ cells/mL) were cultured in the presence or absence of EAA (184.1 ± 1.8 *µ*g/mL) or AAA (165.7 ± 0.6 *µ*g/mL) at its IC_50_ for 72 hours. At the end, cells were fixed with 4% of paraformaldehyde for 30 minutes at 4°C and permeabilized with 0.1% Triton X-100 for 15 minutes. Finally, the cells were stained with 5 *µ*g/mL of Hoechst 33258 dye for 10 minutes at room temperature and visualized and photographed at 100x magnification using Nikon eclipse Ti fluorescence microscope. Nuclei that showed bright blue fluorescence with condensed or fragmented DNA were considered as apoptotic cells, while normal cells appeared to be blue in colour with compact structure [19].

#### 2.12.2. Acridine Orange/Ethidium Bromide Staining

Acridine orange/ethidium bromide (AO/EB) staining is used to determine the nuclear changes associated with apoptosis. In a 12-well plate, the cells (10^5^ cells/mL) were cultured in the presence or absence of EAA (184.1 ± 1.8 *µ*g/mL) or AAA (165.7 ± 0.6 *μ*g/mL) at its IC_50_ for 72 hours. Later, both adherent and cells in suspension were collected and centrifuged at 130 g for 5 minutes. The pellet was resuspended in a solution of 25 *µ*L PBS and 2 *µ*L EB/AO dye (100 *µ*g/mL). Slides were prepared and fluorescence was observed with the help of a Nikon eclipse Ti fluorescence microscope at 200x magnification [20]. AO is a cell-permeant DNA dye that stains both live and dead cells, while EB is cell-impermeant DNA dye that stains only cells with lost membrane integrity. On staining, live cells give green colour with intact membrane and early apoptotic cells display bright green dots in the nuclei as a consequence of chromatin condensation and nuclear fragmentation.

#### 2.12.3. Annexin V/Propidium Iodide (PI) Staining

The annexin V/PI staining is used to discriminate between apoptotic and viable cells. Apoptotic cells are stained positively for annexin V that binds to phosphatidylserine (PS) but are negative for staining with PI, whereas viable cells are negative for both annexin V and PI staining. 10^5^ cells were seeded into each well of a 12-well plate; next day EAA and AAA at its IC_50_ value were added for 72 hours. The subsequent procedures were carried out according to the instructions provided by the manufacturer of ApoDETECTTM Annexin V-FITC Kit as follows, at the end of treatment cells in both monolayer and suspension were collected and centrifuged at 3000 rpm for 1 minute. The supernatant was decanted and the cells washed with 1x PBS and centrifuged at 3000 rpm for 1 minute. The pellet was resuspended in 190 *µ*L of annexin binding buffer and 10 *µ*L of annexin V dye (20 *µ*g/mL) was added, mixed gently, and incubated for 10 minutes at room temperature. The cells were then washed and resuspended in 190 *µ*L of binding buffer and 10 *µ*L of propidium iodide dye (20 *µ*g/mL). Finally, imaging of the apoptotic cells under Nikon fluorescent microscope was done and photographed at 100x [21].

#### 2.12.4. DNA Fragmentation Assay by Agarose Gel Electrophoresis

The endonuclease-mediated cleavage by CAP (caspase activated proteases) enzymes of nuclear DNA leads to formation of DNA fragments of specific size of 180–200 bp, which is one of the evidences of apoptosis. To confirm the apoptotic effect, 10^6^ cells were seeded in a 35 mm dish and treated in presence or absence of EAA and AAA at its IC_50_ value for 72 hours. The protocol for DNA fragmentation assay was as follows: at the end of incubation period, both the cells in suspension and attached cells were pooled together in 1.5 mL Eppendorf vial and centrifuged at 200 g for 10 minutes at 4°C. The pellet was resuspended with 0.5 ml TTE (10 mM Tris-Hcl + 1 mM EDTA + 0.2% Triton-X 100) and vortexed vigorously. The vials were centrifuged at 20,000 g for 10 minutes at 4°C and the supernatant was collected in a new vial. 0.1 mL ice-cold 5 M NaCl and 0.7 mL ice-cold isopropanol were added and vortexed vigorously, followed by overnight incubation at −20°C to allow DNA to precipitate.

Next day, the vials were centrifuged at 20,000 g for 10 minutes at 4°C and washed with 0.5–0.7 mL ice-cold 70% ethanol by centrifuging at 16,000 g for 10 minutes at 4°C. Finally, the pellet was dissolved in 50 *µ*L TE (Tris-Hcl-EDTA). The DNA samples were run on a 1% agarose gel containing ethidium bromide and visualised using a BioRad Gel-Doc system [22].

#### 2.12.5. Quantitative Analysis for DNA Fragmentation

The amount of DNA fragmentation in cancer cells was quantified using diphenylamine method. This method is based on the principle that extensively fragmented double-stranded DNA can be prepared from chromosomal DNA upon centrifugal sedimentation and colorimetrically quantified upon reaction with diphenylamine (DPA), which binds to deoxyribose [22]. 10^6^ cells were seeded in 35 mm dish and treated in presence or absence of EAA and AAA at its IC_50_ values for 72 hours. At the end, 0.5 mL ice-cold extraction buffer (10 mM Tris-Hcl, pH 7.5, containing 1 mM EDTA and 0.2% Triton X-100) was added to cells and centrifuged at 13000 g for 10 minutes at 4°C (tube B). The supernatant was transferred to new vial (tube A) and 0.5 mL of extraction buffer was added to tube B and 0.5 mL of 25% trichloroacetic acid (TCA) to both tubes A and B and vortexed vigorously. The tubes were kept at 4°C for overnight incubation. Next morning, the supernatant was centrifuged for 10 minutes at 13000 ×g and aspirated followed by the addition of 80 *μ*L of 5% TCA to each pellet and the DNA was hydrolyzed by heating for 20 minutes at 83°C in a water bath. Finally, 160 *μ*L of diphenylamine solution was added to each vial. All the tubes were vortexed and then left overnight at room temperature and took the absorbance at 620 nm. The percentage of fragmented DNA was calculated by means of following formula:

(4) $$\begin{array}{c} \%\;\text{fragmented}\;\text{DNA}=\frac{\text{O}.\text{D}\;\text{tube}\;\text{A}}{\text{O}.\text{D}\;\text{tube}\;\text{A}+\text{O}.\text{D}\;\text{tube}\;\text{B}}\times 100. \end{array}$$

### 2.13. Gut Bacterial Hydrolysis

Cytotoxic assessment of plant extracts does not take into account that the naturally occurring compounds may not be active by themselves but may require certain modifications within the parent compound to obtain active molecules which can then act within the body. Thus, a cytotoxicity assay with a prior enzymatic hydrolysis procedure was followed in this study using *β*-glucosidase, an enzyme found not only in bacterial gut flora but also in human body [23]. Briefly, 10^4^ cells/well were seeded in 96-well plate and treated with various concentrations of AAA with and without *β*-glucosidase enzyme treatment for 72 hours. Following this, SRB assay was conducted and percent cytotoxicity calculated using following formula:

(5) %  cell  cytotoxicity=100−{Number  of  viable  cells  Total  number  of  cells×100}.

### 2.14. Gene Expression Studies

In order to evaluate the effects of the AAA extract on the expression of key genes involved in the apoptotic pathway, the total cellular RNA was isolated from the untreated and AAA treated cells (165.7 ± 0.6 *µ*g/mL) using Trizol reagent according to manufacturer's protocol. The integrity and purity of RNA were electrophoretically verified by formaldehyde agarose gel stained with ethidium bromide and the O.D_260 nm_/O.D_280 nm_ ratio was quantified using nanodrop. One microgram (1 *µ*g) of RNA was reverse transcribed into cDNA in a reverse transcription reaction mixture containing 1x PCR buffer, 0.5 mM deoxynucleoside triphosphates (dNTPs), 2.5 *µ*M of oligo d (T) primer, and 2.5 units of MuLV reverse transcriptase [24]. The reaction mixture was incubated at 46°C for 1 hour. The synthesised cDNA was quantified using nanodrop and equal amount of cDNA was used in the present study to quantify the amount of change in expression of cancer cells after treatment. PCR reactions was carried out in a final volume of 20 *µ*L containing 12.5 *µ*L of PCR green mix (Fermentas), 1 *µ*g cDNA, 1 *µ*L of forward and reverse primer (1 mM of each primer), and 4.5 *µ*L of nuclease free water. The PCR products were run on 1.8% agarose gel using ethidium bromide, and densitometry scanning of the bands was done using an Alpha Innotech Gel Doc using the Alpha imager EP software. The values for each gene product were normalised to the house-keeping gene, *β*-actin. The primer sequences of the genes included in our study are shown in Table S1 in Supplementary Material available online at http://dx.doi.org/10.1155/2014/129697 [25–28].

### 2.15. Cell Cycle Analysis by Flow Cytometry

If any treatment results in the slowing down of the cell cycle, then the number of cells in the various phases of the cell cycle would vary from that of the control cells. To check the effect of AAA on cell cycle distribution of COLO-205 cells, flow cytometric analysis by PI staining was performed. PI is a highly water soluble, fluorescent dye that binds to DNA by intercalating between the bases in double stranded nucleic acids of exposed nuclei. The relative fluorescence intensity of PI is an indirect measure of the cellular DNA content [29]. 2 × 10^6^ cells were seeded in a 60 mm dish and treated with IC_50_ value of AAA (165.7 ± 0.6 *µ*g/mL) for 72 hours. Later, cells in suspension along with monolayer cells which were detached by trypsinization and were pooled and centrifuged at 300 g for 5 minutes. To the pellet, we added 0.5 mL of PBS and fixed the cells by transferring 4.5 mL of 70% ethanol to cell suspension (added in a dropwise manner) for at least 2 hours at −20°C. The cell solution was then centrifuged at 300 g for 5 minutes and the pellet was resuspended in 1 mL of PBS and stained with 1 mL of PI staining solution (100 *µ*g/mL RNAase, 50 *µ*g/mL propidium iodide, and 0.5% Triton-X 100 in PBS) for 30 minutes at room temperature in the dark. Samples were analyzed using BD AccuriR C6 Flow Cytometer in the FL2 channel (laser 488 nm and filter 485/40 nm). 10,000 events per sample were recorded for each experiment.

### 2.16. GC-MS Analysis

GC-MS analysis was carried out using thermo GC model TRACE 1300 and thermo MS model TSQ 8000 (triple quadrapole) equipment employing the following conditions: Thermo TG 5MS Column having dimensions of “30 m × 0.25 mm × 0.25 *µ*m”, operating in electron impact mode at 70 eV. Helium (99.999%) was used as carrier gas at a constant flow rate of 1 mL per minute and an injection volume of 1 *μ*L. An injector temperature of 250°C and an ion-source temperature of 280°C were employed. The Oven program consisted of maintaining the temperature at 60°C for 2 minutes followed by an increase to 250°C at the rate of 15°C/minute and maintaining this temperature of 250°C for a further 15 minutes. The percentage of each chemical constituent was calculated by comparing the average peak area to the total areas.

### 2.17. Statistical Analysis

All experiments were performed thrice in triplicate and the data was presented as mean ± SD. Statistical analysis was conducted with the GraphPad InStat software. One way ANOVA was used to compare untreated cells with treated cells in case of* in vitro* studies. A minimum *P* value < 0.05 was considered to indicate a statistically significant difference.

## 3. Results

### 3.1. Total Phenolic Content

The results showed that EAA contained greater amounts of phenolic compounds with a TPC value of 419.2 ± 1.3 *µ*g GAE/gram of dry extract as compared to AAA having 273.8 ± 2.5 *µ*g GAE/gram of dry extract of total phenolic content.

### 3.2. *In Vitro* Antioxidant Activity

The capacity of an extract to scavenge free radical formation was examined by performing two different assays, that is, FRAP and DPPH assay. The results showed EAA to exhibit greater antioxidant activity with a FRAP value of 195 ± 1.2 *µ*M Fe (II)/gram of dry extract and IC_50_ value of DPPH radical of 288.3 ± 1.4 *µ*g/mL of dry extract as compared to AAA with FRAP value of 121 ± 1.5 and IC_50_ value of DPPH radical 372.6 ± 1.7. A positive corelation was found between TPC and antioxidant activity in both extracts with an *R* ^2^ value of 0.998.

### 3.3. Cell Viability Assay

Live cells have intact cell membrane that excludes trypan blue dye, whereas dead cells do not. As clearly evident in Figure S1, AA treatment of COLO-205 cells leads to a dose and time dependent decrease in cell viability as compared to the untreated controls. Figure S1 A represents the percent viability of EAA treated COLO-205 cells which decreased to 35.49 ± 1.1 (*P* < 0.01) and 25.34 ± 0.8 (*P* < 0.001) at 48 and 72 hours of treatment, respectively, at a dose of 200 *µ*g/mL. A similar trend was observed but was more pronounced in the AAA treated COLO-205 cells as is seen in Figure S1 B with percent viability decreasing to 19.01 ± 0.2 (*P* < 0.0001) at 72 hours and 30.45 ± 0.6 (*P* < 0.001) at 48 hours. Since we observed more marked effects at 72 hours of treatment, subsequent experiments were performed at this time period.

### 3.4. Cell Cytotoxicity by MTT Assay

**Figure 2:**
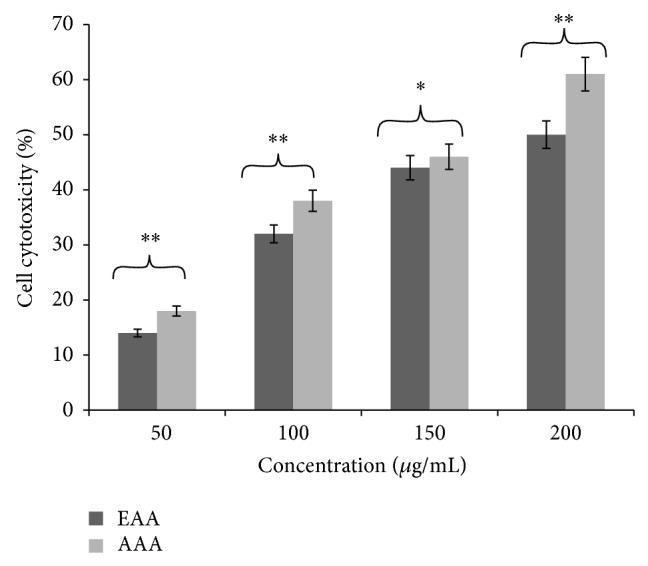
Dose dependent cytotoxic effect of AA extracts on COLO-205 cells by MTT assay. Cells were incubated with various concentrations of AA extracts (50, 100, 150, and 200 *μ*g/mL) for 72 hours. Data presented as mean ± SD (*n* = 3) and compared as percent cytotoxicity of EAA versus percent cytotoxicity of AAA. ^*^ *P* < 0.05; ^**^ *P* < 0.01.

In Figure 2, the percent cell cytotoxicity of EAA and AAA towards COLO-205 cells revealed that AAA was relatively more cytotoxic towards COLO-205 cells as evidenced from the lower IC_50_ of 165.7 ± 0.6 *µ*g/mL which was the concentration that caused 50% loss of cell viability, as compared to EAA with an IC_50_ of 184.1 ± 1.8 *µ*g/mL. The IC_50_ of each of the AA root extracts against COLO-205 cells was used as the effective dose for our next set of experiments.

### 3.5. Assessment of Growth Kinetics

**Figure 3:**
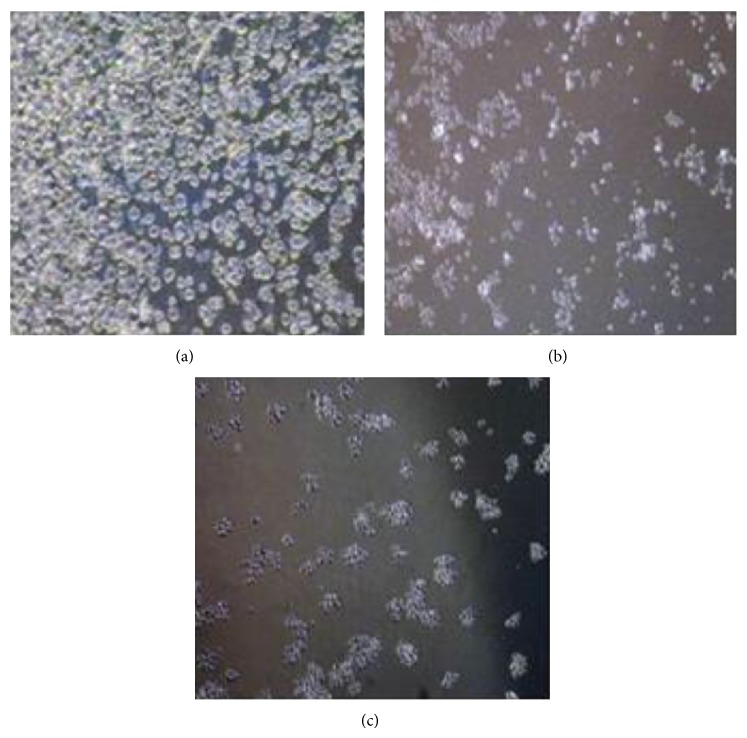
AA extracts lead to growth inhibition in COLO-205 cells. To study the effect of AAA and EAA on cell growth; COLO-205 cells were treated with IC_50_ value of the extracts and kept for a period of 72 hours. (a) Control, (b) EAA, and (c) AAA after 72 hours (magnification at 100x).

Exponentially growing cells, when treated with IC_50_ of EAA (184.1 ± 1.8 *µ*g/mL) and AAA (165.7 ± 0.6 *µ*g/mL) for 72 hours, showed marked regression in growth, which was evident when viewed under a phase contrast microscope. The loss of cellular attachment to the substratum and increase in number of floating cells seen in the cell culture wells were indicators of cell death and revealed altered growth kinetics in treated cells.  Figure 3(a) showed the normal confluence of untreated control culture of COLO-205 cells at 72 hours with no apparent signs of cell death, while after treatment marked decrease in cell number was evident owing to cell death (Figures 3(b) and 3(c)) and this effect was more pronounced after AAA treatment indicating enhanced growth inhibitory effects of AAA towards COLO-205 cells in contrast to EAA.

### 3.6. *In Vitro* Wound Scratch Assay

**Figure 4:**
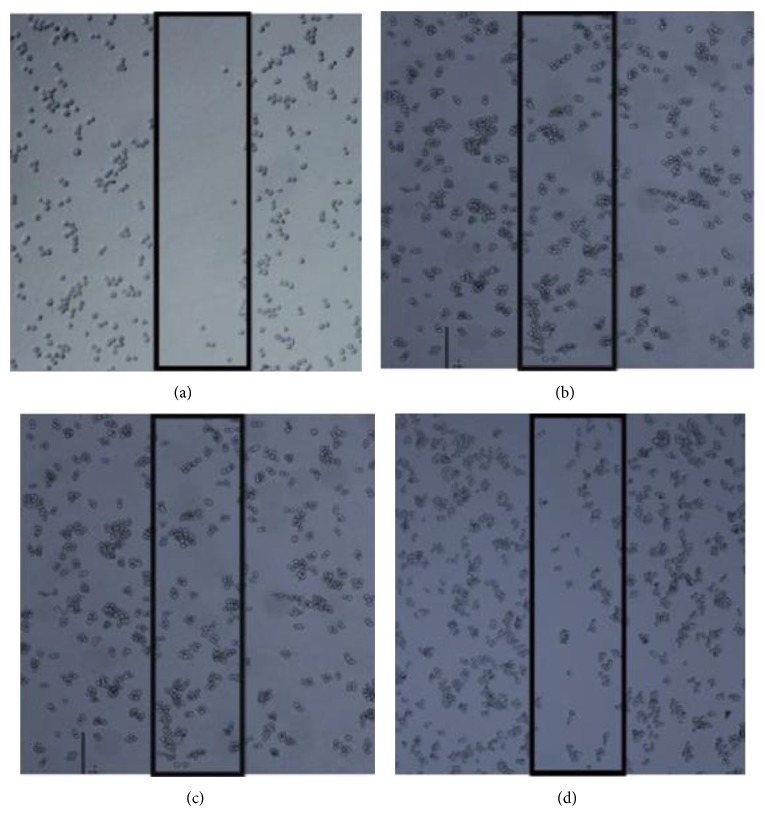
Inhibitory effects of AA extracts on the migration of COLO-205 cells. (a) Initial view of scratch at time 0, prior to any treatment, (b) control, after 24 hours, (c) EAA treated cells at 24 hours, and (d) AAA treated cells at 24 hours.

The ability of EAA and AAA to inhibit cell motility was investigated by* in vitro* scratch assay. A denuded area was created and cells were exposed to the IC_50_ concentration of AAA (165.7 ± 0.6 *μ*g/mL) and EAA (184.1 ± 1.8 *μ*g/mL) for 24 hours.  Figure 4(a) showed a representative scratch at time 0 of wound initiation. After 24 hours the cells present in the control well (Figure 4(b)) were able to divide and repopulate the space created by the scratch. However, treatment of the cells with both of the AA extracts leads to a decrease in the rate at which the cells were able to divide and thereby migrate into the clear area created by the scratch. Inhibition of wound reepithelialization by AAA extract was more evident through pictorial image as compared to EAA (Figure 4(c)) thereby indicating greater inhibitory effects on cell proliferation leading to much lower migration of cells (Figure 4(d)).

### 3.7. Clonogenic Cell Survival Assay

**Figure 5:**
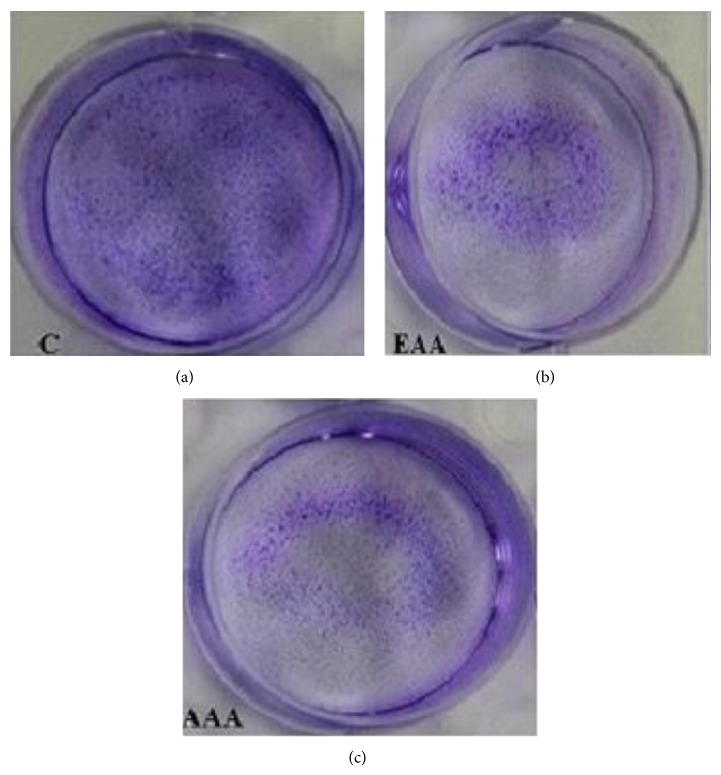
Colony formation inhibition of COLO-205 cells on treatment with AA extracts for 14 days. Cells were fixed with 4% paraformaldehyde and stained with crystal violet. “C” control untreated well “EAA” and “AAA” represents the treated wells.

A significant decrease in number of colonies present in the treated wells, after an incubation period of 2 weeks, was observed compared to the untreated control well (Figure 5). AAA treated cells at a concentration of 165.7 ± 0.6 *µ*g/mL showed 42.1 ± 1.3 percent decrease in colony forming ability, while EAA treatment at its IC_50_ (184.1 ± 1.8 *µ*g/mL) showed 56.2 ± 0.9 percent reduction in comparison with untreated control which was taken as 100 percent colony forming ability.

### 3.8. Antiproliferative Assay

**Figure 6:**
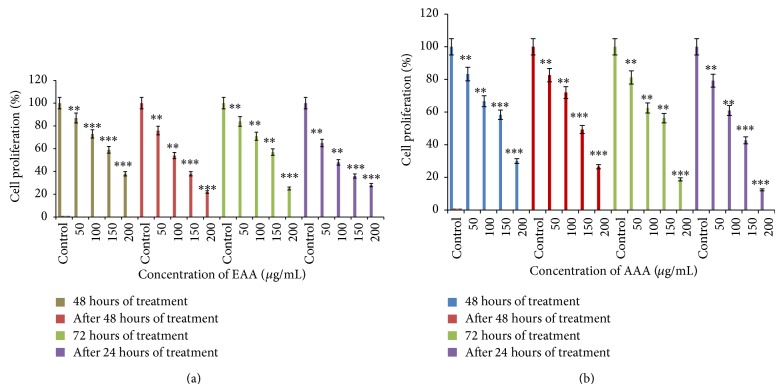
Antiproliferative effect of AA extracts on COLO-205 cells: (a) EAA and (b) AAA. Data presented as percent proliferation of treated cells (*n* = 3) compared to untreated control cells. ^*^ *P* < 0.05, ^**^ *P* < 0.01, and ^***^ *P* < 0.001.

To evaluate the effect of AA extracts on proliferation, a direct cell count was performed. We observed that both of the extracts lead to cell killing when they were present in the medium. However, on renewal with fresh medium, both EAA and AAA treated cells responded in different modes. The cytostatic effects were seen in EAA treated cells as displayed in Figure 6(a). The effects were more pronounced at the highest concentration and were also time dependent with 38.5 ± 0.3 and 22.6 ± 0.3 percent proliferation after 48 hours and after 48 hours of medium replacement, respectively, whereas 24.9 ± 0.3 and 27.7 ± 0.3 percent reduced proliferation were seen after 72 hours and after 24 hours of medium replacement treatment, at 200 *µ*g/mL.  Figure 6(b) revealed the cytocidal nature of AAA towards COLO-205 cells, conferring an irreversible action on the cell cycle, with 30.3 ± 1.8 and 26.6 ± 1.4 percent proliferation after 48 hours and after 48 hours of medium replacement, respectively, whereas 18.7 ± 1.4 and 14.3 ± 1.7 percent proliferation were seen after 72 hours and after 24 hours of medium replacement treatment at a concentration of 200 *µ*g/mL.

### 3.9. Induction of Apoptosis

**Figure 7:**
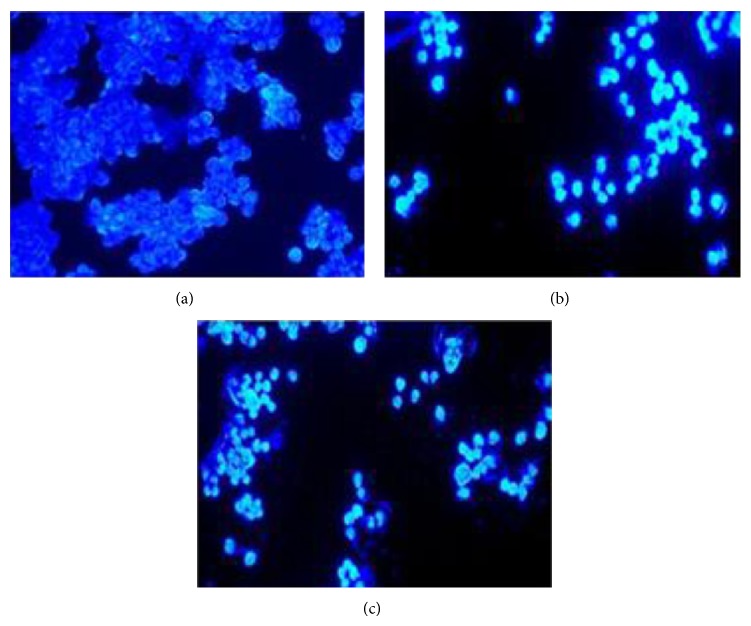
Evaluation of apoptotic changes in COLO-205 cells by Hoechst 33258 staining. (a) Control, (b) EAA, and (c) AAA (magnification 100x).

The morphological changes in cell nuclei were determined by fluorescence microscope by staining cells with Hoechst 33258 dye. The morphological observation in the cell nuclei of COLO-205 cells after treatment with or without AA root extracts for 72 hours is shown in Figure 7. The untreated cells appeared to be intact and round in shape and the nuclei were stained with a less bright blue fluorescence (Figure 7(a)), whereby cells treated with AA extracts showed evidence of cell shrinkage and chromatin condensation with bright blue fluorescence. However, AAA treatment at its IC_50_ value (Figure 7(c)) exhibited greater evidence of apoptosis with more pronounced chromatin condensation and segregated bodies as compared to EAA (Figure 7(b)).

Apoptotic cells were also identified by AO/EB and annexin V/PI staining. Untreated control cells displayed normal green nucleus (Figure S2 A) while for the same field (Figure S2 B) there was no evidence of red fluorescence as ethidium bromide (EB) is only taken up by cells when cytoplasmic membrane integrity is lost, pointing to the fact that the cells were live. Similar results were also seen in the untreated control cells stained with annexin V/PI (Figure S3 A-B). The cells treated at the IC_50_ of AAA (165.7 ± 0.6 *μ*g/mL) for 72 hours showed marked changes in morphology such as irregular shape, membrane blebbing, and condensed and fragmented chromatin to produce apoptotic bodies with enhanced levels of fluorescent nuclei as observed in Figure S2 E-F and externalisation of phosphatidylserine leading to binding of annexin V conjugated to FITC with signs of both early (Figure S3 E) and late stage apoptosis (Figure S3 F). All these effects were more pronounced in the AAA treated cells as compared to those treated with EAA (Figure S2-3 C-D).

**Figure 8:**
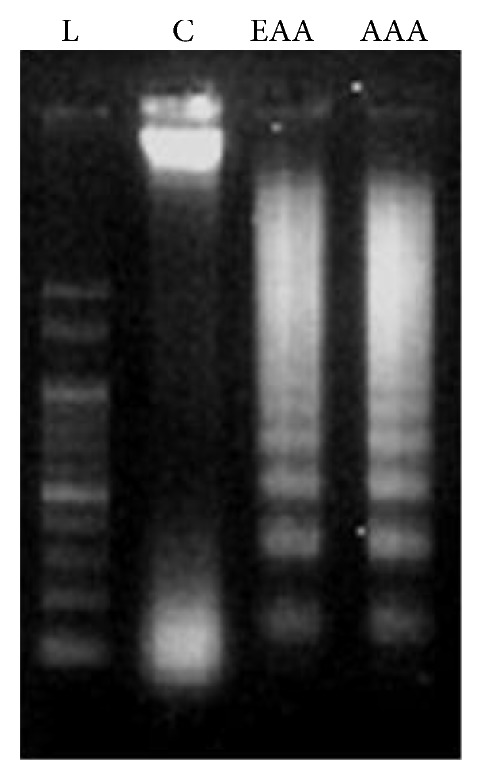
AA extracts lead to induction of DNA fragmentation in the COLO-205 cells. Lane 1: 100 bp ladder, lane 2: untreated control cells, lane 3: EAA treated cells, and lane 4: AAA treated cells.

The ladder like DNA fragmentation is qualitative indicator of apoptosis. No evidence of fragmentation was seen in the control lane marked C (Figure 8). A ladder like pattern of damaged DNA was observed after 72 hours of treatment with IC_50_ of AAA and EAA. To quantify the degree of DNA fragmentation after 72 hours of treatment the DPA method revealed an increased percentage of fragmentation in AAA treated cells of 35 ± 2.3%, while EAA treatment leads to 18 ± 2.1% in contrast to untreated cells having only 8 ± 1.2% fragmented DNA.

### 3.10. Gut Bacterial Hydrolysis

The growth inhibitory effect of AAA over EAA was clearly evident from the above results. Therefore, AAA was further subjected to gut bacterial hydrolysis, to check whether it is inherently cytotoxic or acts as a prodrug, which needs some modification to its original form to become fully active within the human body. Treatment of the various concentrations of the AAA extract with metabolic enzyme prior to cytotoxicity testing showed an increased percent cytotoxicity which was significant. The most significant effect was seen at 250 *µ*g/mL, wherein the cytotoxicity increased from 67.1 ± 1.2 to 79.2 ± 1.6 percent (*P* < 0.001). The inference drawn from this result indicates that AAA is behaving as a prodrug, which needs to be further acted upon by enzymes present within the body to enhance its cytotoxicity within the system (Figure S4).

### 3.11. Gene Expression Studies

**Figure 9:**
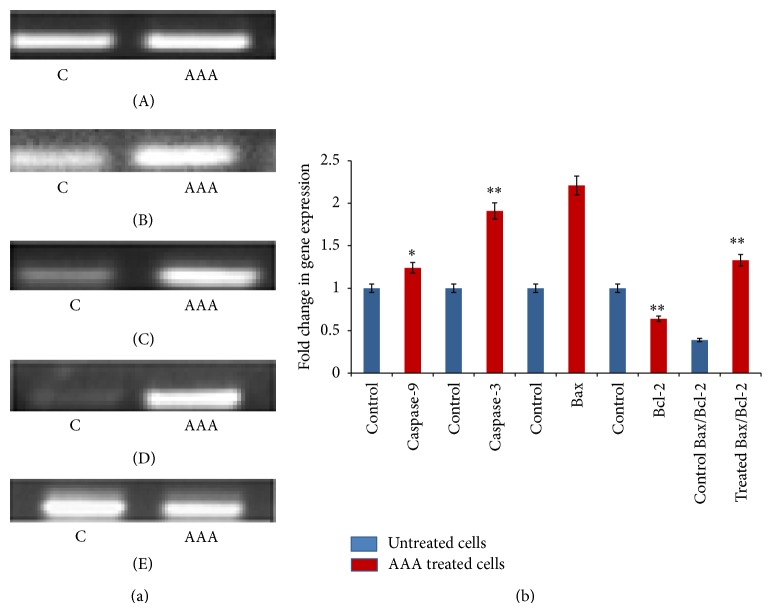
(a) AAA induced activation of intrinsic apoptotic pathway in COLO-205 cells. Agarose gel electrophoresis of PCR products of (A) *β*-actin, (B) caspase-9, (C) caspase-3, (D) Bax, and (E) Bcl-2 genes. (b) The fold change in expression of apoptosis-associated gene after AAA treatment. The densitometric analysis was done of the PCR products of caspase-9, caspase-3, Bax, and Bcl-2 genes to measure the fold change in each gene and was normalised to the house-keeping gene *β*-actin. Data presented as fold change in gene expression between AAA treated cells and untreated control cells. ^*^ *P* < 0.05; ^**^ *P* < 0.01.

RT-PCR analysis revealed a significant upregulation in the expression of proapoptotic genes such as caspase-3, caspase-9, and Bax in the AAA treated cells as compared to control. At the same time, expression of the antiapoptotic Bcl-2 gene was downregulated upon treatment of COLO-205 cells with AAA at its IC_50_ value of 165.7 ± 0.6 *µ*g/mL. The fold change in gene expression between untreated and AAA treated cells after 72 hours was calculated and an increment in the expression of caspase-9 from 1.0 to 1.24 (*P* < 0.05) with a concomitant increase in the level of caspase-3 which is the executioner caspase, from 1.0 to 1.9 (*P* < 0.01). An increase in the ratio of Bax/Bcl-2 in the AAA treated cells to 1.33 from 0.39 in the untreated controls (*P* < 0.01) was seen which signifies propensity towards apoptosis (Figures 9(a) and 9(b)). These results point towards the involvement of the intrinsic pathways of apoptosis induced by the AAA in COLO-205 cells.

### 3.12. Cell Cycle Analysis

**Figure 10:**
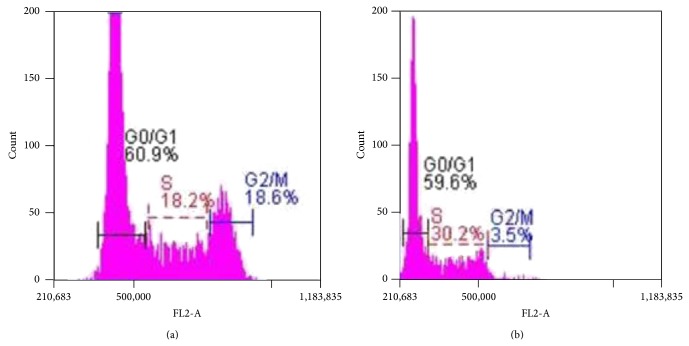
AAA leads to S phase arrest in COLO-205 cells. (a) Histogram of cell cycle distribution of control untreated cells. (b) Histogram of cell cycle distribution of AAA treated cells as assessed by flow cytometry.

**Figure 11:**
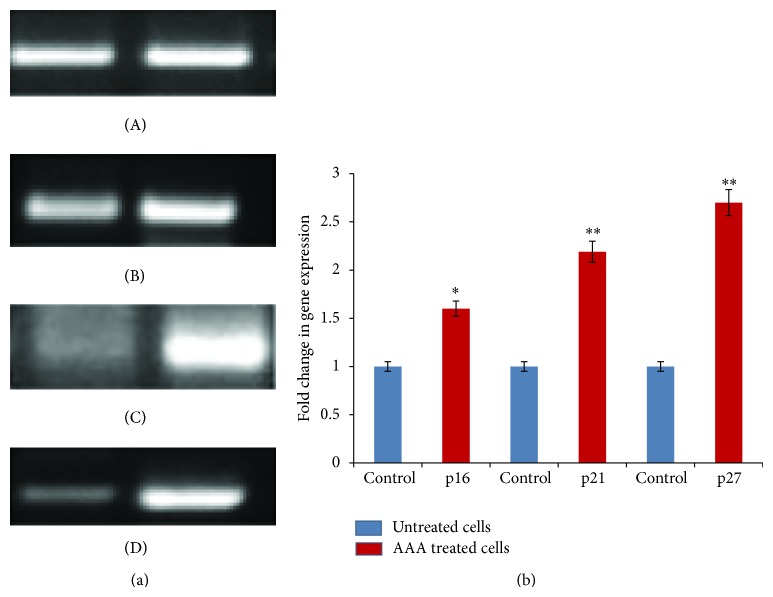
(a) CKI upregulation by AAA induces cell cycle arrest in COLO-205 cells. Agarose gel electrophoresis of PCR products of (A) *β*-actin, (B) p16, (C) p21, and (D) p27 genes. (b) The fold change in expression of CKI's gene after AAA treatment. The densitometric analysis was done of the PCR products p16, p21, and p27 gene to measure the fold change in each gene and was normalised to house-keeping gene *β*-actin. Data presented as fold change in gene expression between AAA treated cells and untreated control cells. ^*^ *P* < 0.05; ^**^ *P* < 0.01.

Cell cycle phase distribution was analyzed by flow cytometry with PI staining and the percentage of cells in G_0_/G1, S, and G2/M phases was calculated using CFlow Plus software. Histograms of the flow cytometric data are shown in Figure 10. In untreated cells, 60.9 ± 1.9, 18.2 ± 1.7, and 18.6 ± 2.1 percent of the cells were distributed among G_0_/G1, S, and G2/M phase, respectively. Exposure of cells to AAA (165.7 ± 0.6 *µ*g/mL) for 72 hours resulted in cell accumulation at S phase with 59.6 ± 1.4, 30.2 ± 2.1, and 3.5 ± 1.6 percent distribution in G_0_/G1, S, and G2/M phase, respectively. This arrest was accompanied by increase in levels of p16, p21, and p27 as compared to untreated controls from 1 to 1.4 (*P* < 0.05), 1 to 2.1 (*P* < 0.01), and 1 to 2.6 (*P* < 0.01) (Figures 11(a) and 11(b)), which are inhibitors of cell cycle and could provide an explanation of cell cycle inhibition.

### 3.13. GC-MS Analysis

GC-MS chromatogram of the AAA extract showed ten peaks indicating the presence of ten chemical constituents. On comparison of the mass spectra of the constituents with the NIST library the ten constituents were characterized and identified (Figure S5). The total MS running time was 32 minutes. The active principles with their retention time (RT), molecular formula, and peak area (%) in AAA extracts are presented in Supplementary Table S2.

## 4. Discussion

Plants have always represented a rich source of compounds that possess unique medicinal properties. The benefit of herbs already exists in the ancient literature of Ayurveda and Unani medicine [6]. Various secondary metabolites are known to act as anticancer agents [30, 31]. Therefore, AAA and EAA containing these compounds may serve as a potential source of bioactive compounds in the treatment of colon cancer. The TPC values were found to be more in EAA than AAA. Also, the results from antioxidant assay showed EAA to possess greater capability to scavenge the free radicals in comparison to AAA, suggesting greater proportion of phenolics in EAA which are known to act as free radical scavengers. Others have also documented the positive correlation between free radical scavenging activity and TPC value [32, 33] and similar observation was seen in our study. The cell viability assay was performed to determine the effect of AA root extracts on viability of COLO-205 cells. The results showed a dose and time dependent response in growth inhibition which was more pronounced in AAA and was in agreement with the MTT assay. Although antioxidants have been linked to cytotoxicity, we observed that AAA was more cytotoxic than EAA, even though EAA possessed higher TPC and antioxidant activity. These findings suggested the presence of active compounds other than phenolics present in AAA that may be responsible for its cytotoxic effect. Indeed, previous phytochemical investigations have suggested that roots of AA are rich in saponins, which could explain greater cytotoxic effect in AAA treated cells [33, 34]. In addition in our previous studies to ascertain selectivity of CAM therapies towards cancer cells, we have demonstrated that AA root extracts display no significant cytotoxicity on normal epithelial cells [35]. These results, taken together, point the specificity towards COLO-205 cells and the effectiveness of CAM therapies over conventional ones.

Wound healing is a cellular process which allows cells to restore their original architecture and function in their wound site [17]. This property of malignant cells was reversed on application of EAA and AAA, wherein treatment with AAA was able to halt the process of migration to a greater extent in comparison to EAA, indicating that AAA has the potential to prevent metastasis. Clonogenic cell survival assay was used in our study to determine antiproliferative effects of AA extracts by checking its ability to inhibit cancer cells from dividing and forming colonies. AAA treated cells were shown to exhibit greater effect on colony formation over EAA treated cells indicating that AAA possesses certain botanical compounds which can impinge upon the cell cycle leading to arrest of cell growth. Reduction in growth of cancer cells after treatment with various plant extracts was also observed by other researchers, indicating alterations in growth kinetics of cancer cells [36].

The assessment of proliferative behaviour of cells is a noteworthy step as most of the anticancer drugs are less cytocidal to quiescent cells than to actively proliferating cells [37]. Antiproliferative effect of any agent/extract can have 2 possible outcomes on cell growth. When the extract stops or delays the cell from dividing, it said to be cytostatic and this may be reversible thereby allowing a cell to undergo multiplication in fresh medium even after the specific treatment. The second category highlights the cytocidal/irreversible nature of an extract, which involves killing of cells with no further increase in cell number even after removal of medium containing the extract [38]. The results of the proliferation assay have thrown some interesting light on the mechanism of action of AA. The AAA treated cells significantly showed greater cytocidal effect thereby hindering the multiplication of the cells in an irreversible manner in comparison with the cytostatic EAA treated cells at their highest concentration, after 72 hours of treatment. Our results were in agreement with a number of plant based botanicals which form a part of traditional medicines which acting in a similar manner either make the cells undergo apoptosis or make the cells exit the cell cycle and into the G_0_ stage. Curcumin, a diarylheptanoid derivative of turmeric, has also been attributed in suppression of cell proliferation by altering the cell cycle in various* in vitro* colon cancer models [39].

The antiproliferative and cytotoxic effects of AA root extracts are likely to be linked via activation of apoptosis. The cell death was identified by a phase contrast microscopy, which revealed abundance of morphological alterations including loss of cell viability, irregular shape, and cellular detachment in AAA treated cells as compared to EAA treated cells. The apoptotic cell death in treated cells was observed by different assays indicating characteristic leading to cell death. Studies on plant extract mediated apoptosis point towards the role of the intrinsic pathway.* Euphorbia formosana* root extract induced the intrinsic pathway in THP-1 human leukemic cells [40]. Increased expression of the proapoptotic protein (Bax), along with reduced level of the antiapoptotic protein (Bcl-2), was observed in breast cancer MCF-7 cells after treatment with* Lethariella zahlbruckneri* extract [41]. In our investigation we too observed increased levels of caspase-9, caspase-3, and Bax, along with decreased expression of Bcl-2 in AAA treated cells, thereby confirming the induction of apoptosis in COLO-205 cells via mitochondrial pathway.

An altered regulatory mechanism prevalent in the cell cycle plays pivotal role in the growth of various types of cancer cell and is an important target in cancer therapy. The aqueous extracts of AA were able to induce their antiproliferative activity as seen in the form of antimigration and inhibition of colony formation properties in part, by deregulating the cell cycle as revealed by an increase in the percentage of cells in the S phase. For a cell to be able to divide into two daughter cells the synthesis and duplication of the DNA are an absolute requirement and any abnormality in this would lead to a halt in the cell cycle progression. The phenomenon of DNA damage is a continuous setback that cells must deal with and impacts their viability and proliferation. Failure to undergo the repair to damaged DNA generally leads to alterations in genome resulting in multistep mutations leading to carcinogenesis [42]. Several checkpoints have been employed by the cell to identify and repair the DNA damage by halting/stalling their progress through the various phases of the cell cycle such as G_0_/G1, S, and G2/M checkpoints. We observed arrest at S phase of cell cycle upon AAA treatment leading to a halt in cell cycle progression. A number of CAM sources have exhibited significant inhibitory effects on cancer cells via disruption of cell cycle progression. Amongst various traditional Chinese medicines (TCM),* Ganoderma* is the one of commonly used herbal medicine in Asia. Studies have shown that treatment with the chloroform extract of* Ganoderma* causes cell cycle arrest in cells [43]. Growth inhibitory effect of celery seed extracts on human gastric cancer BGC-823 cells was determined, which was associated with cycle arrest in the S phase and decreased levels of cyclin A and CDK-2 [44]. The crude water extract of* Centella asiatica* showed S and G2/M arrest in human colon adenocarcinoma-derived Caco-2 cells, accompanied with accumulation of cyclin B1 protein in the cells [45].

In order to get a clearer insight into the underlying mechanism for cell cycle arrest by AAA, the regulatory genes of the cell cycle were evaluated, namely, the cyclin dependent kinase inhibitors, or CKIs, p16, p21, and p27 genes. An upregulation of p16, p21, and p27 level in AAA treated cells indicated stimulatory role of AAA in activation of inhibitors of cell cycle progression thereby causing an arrest in cell cycle process. Several plants have shown the role of CKI in inhibition of cancer cell progression. An increase in p53, p21WAF1/Cip1, and p27Kip1 levels, indicators of cell cycle arrest, was observed in murine B16F10 melanoma cells on treatment with* Moringa oleifera *Lam.*, Eremomastax speciosa *(Hochst.) Cufod., and* Aframomum melegueta *K. Schum. extracts. [46]. DATS, a garlic-derived organosulfur compound, showed growth inhibitory effects on pancreatic cancer cells (Capan-2) through elevated levels of cyclin B1 and p21 and reduced levels of cyclin D1 [47].

For investigation of volatile biological compounds present in AAA extract, GC-MS analysis was done and the mass spectra revealed the presence of 10 compounds. The spectrum of the unknown component was compared with the spectrum of the known components stored in the NIST library. Hence, the identified phytocomponents using GC-MS can be further isolated and studies on their biological activity in an* in vitro* system can be done.

## 5. Conclusion

In conclusion, our findings indicate that the aqueous extract of* Achyranthes aspera* suppresses the cell proliferation and increases cytotoxicity in a dose and time dependent manner in colon cancer cells. This ability of AAA could be attributed to the induction of apoptosis via the mitochondrial-mediated pathway and arresting the cells in S phase of cell cycle in COLO-205 cells. However, the exact mechanisms and the molecular determinants that are responsible for the initiation of apoptosis need to be addressed. Thus, further studies on AAA as a novel therapeutic agent having anticancer activity, originating from plants, are warranted.

## Supplementary Material

Supplementary Material: To assess the cytotoxic effects of aqueous(AAA) and ethanolic(EAA) extracts of Achyranthes Aspera Root on human colon cancer cells (COLO-205) various assays were carried out.
The results revealed that both the extracts were able to reduce the viability in a dose dependent manner although AAA had more pronounced effects (Figure S1).These results were qualitatively verified by AO/EB(Figure S2) and Annexin V/PI(Figure S3) staining which revealed the ability of both the extracts to induce apoptosis in the COLO-205 cells, whereas the untreated cells showed no evidence of apoptosis.
The data generated revealed that in comparison to EAA, AAA showed more promise in terms of eliciting cell death. Therefore this extract was pre-treated with *β*-glucosidase to mimic in vivo conditions and it was seen that enhanced cytotoxicity to the COLO-205 was observed as compared to the extract in which no pre-treatment with the enzyme had been carried out(S4). These results indicated that the AAA could be modified by enzymes present in vivo leading to greater toxicity to the cancer cells.
Finally GC-MS analysis of AAA revealed the presence of 10 biological compounds which could be responsible for the various effects seen on COLO-205 cells (S5).
