# Supplementary material for: Achyranthes aspera Root Extracts Induce Human Colon Cancer Cell (COLO-205) Death by Triggering the Mitochondrial Apoptosis Pathway and S Phase Cell Cycle Arrest
Source: ScientificWorldJournal. 2014 Oct 27;2014:129697. doi: 10.1155/2014/129697 (PMC4225856; doi:10.1155/2014/129697)
Supplement: Supplementary file 1 — Supplementary Material: To assess the cytotoxic effects of aqueous(AAA) and ethanolic(EAA) extracts of Achyranthes Aspera Root on human colon cancer cells (COLO-205) various assays were carried out. The results revealed that both the extracts were able to reduce the viability in a dose dependent manner although AAA had more pronounced effects (Figure S1).These results were qualitatively verified by AO/EB(Figure S2) and Annexin V/PI(Figure S3) staining which revealed the ability of both the extracts to induce apoptosis in the COLO-205 cells, whereas the untreated cells showed no evidence of apoptosis. The data generated revealed that in comparison to EAA, AAA showed more promise in terms of eliciting cell death. Therefore this extract was pre-treated with β-glucosidase to mimic in vivo conditions and it was seen that enhanced cytotoxicity to the COLO-205 was observed as compared to the extract in which no pre-treatment with the enzyme had been carried out(S4). These results indicated that the AAA could be modified by enzymes present in vivo leading to greater toxicity to the cancer cells. Finally GC-MS analysis of AAA revealed the presence of 10 biological compounds which could be responsible for the various effects seen on COLO-205 cells (S5). [file 129697.f1.docx]

**Supplementary Tables**

**Tables**

**Table S1**: **List of genes used in the study and their cycling parameters**

| **Gene** | **PCR Programme** | **Reference** |
| --- | --- | --- |
| **β-actin**  F‘5AGCCGTGGCCATCTCTTGCTCGAAG3’  R‘5 GCCATGTACGTTGCTATCCAGGCTG 3’ | 94^0^C-4 min, 34 cycles of 94^0^C-30 sec,  65^0^C-30 sec and 72^0^C-1min and final  extension at 72^0^C-10 min  **Product Size**- 300 bp | [25] |
| **Caspase-9**  F‘5TGTCCTACTCTACTTTCCCAGGTTTT3’  R-‘5GTGAGCCCACTGCTCAAAGAT 3’ | 95^0^C-5 min, 40 cycles of 95^0^C -45 sec, 60^0^C-1min and 72^0^C-1min and final extension at 72^0^C-10 min  **Product Size**- 101 bp | [26] |
| **Caspase-3**  F‘5CAGTGGAGGCCGACTTCTTG3’ R‘5TGGCACAAAGCGACTGGAT3’ | 95^0^C-5 min, 40 cycles of 95^0^C-45 sec, 60^0^C-1min and 72^0^C-1min and final extension at 72^0^C -10 min  **Product Size**- 102 bp | [26] |
| **Bax**  F ‘5TCCACCAAGAAGCTGAGCGA3’  R‘ 5 GTCCAGCCCATGATGGTTCT3’ | 95^0^C-5 min, 40 cycles of 95^0^C-45 sec,  65^0^C-1min and 72^0^C-1min and final  extension at 72^0^C-10 mins  **Product Size**- 257 bp | [27] |
| **Bcl-2**  F-5'GTGGAGGAGCTCTTCAGGGA3'  R- 5'AGGCACCCAGGGTGATGCAA3' | 93^0^C-2min, 40 cycles of 92^0^C-30 sec,  55^0^C-40 sec and 72^0^C-30 sec and final extension at 72^0^C-10 min.  **Product Size**- 304 bp | [28] |
| **p16**  F-‘5ATCTGATCTCCATCGCAGGG3’  R- ‘5AAACTGTGCTCCTCCCCTAC3’ | 93^0^C-3min, 38 cycles of 93^0^C-30 sec,  59^0^C-45 sec and 72^0^C- 60 sec and final extension at 72^0^C-10 min.  **Product Size**  236 bp | Primer 3  software |
| **p21**  F-‘5GTTCTACCTCAGGCAGCTCA3’  R-‘5AATGAACTGGGGAGGGATGG3’ | 93^0^C-3min, 38 cycles of 93^0^C-30 sec,  59^0^C-45 sec and 72^0^C- 60 sec and final extension at 72^0^C-10 min.  **Product Size**  208 bp | Primer 3  software |
| **p27**  F-‘5GCAAGTACGAGTGGCAAGAG3’  R-‘5GTCGCTTCCTTATTCCTGCG3’ | 93^0^C-3min, 38 cycles of 93^0^C-30 sec, 56^0^C-45 sec and 72^0^C- 60 sec and final extension at 72^0^C-10 min.  **Product Size**  249 bp | Primer 3 software |

**Table S2: GC-MS Spectra of AAA extract.**

| **Compound name** | **Retention time  (Minutes)** | **% Peak Area** | **Molecular Weight** |
| --- | --- | --- | --- |
| Benzyl Benzoate | 17.84 | 6.36 | C_14_H_12_O_2_ |
| Benzoic acid, 2-phenylhydrazide | 18.72 | 3.26 | C_13_H_12_N_2_O |
| 2,2,4-Trimethyl3-(3,8,12,16tetramethylheptadeca-3,7,11,15-tetraenyl)-cyclohexanol | 23.64 | 13.17 | C_30_H_52_O |
| Glycine,  N-[(3à,5á,7à,12à)-24-oxo-3,7,12-tris-[(trimethylsilyl)-oxy]-cholan-2-4-yl], methyl ester | 24.10 | 3.60 | C_36_H_69_NO_6_Si_3_ |
| Cyclodecasiloxane, eicosamethylOctasiloxane, | 25.72 | 3.05 | C_20_H_60_O_10_Si_10_ |
| [5-(3Methoxymethoxy-10,13-dimethyl 2,3,4,9,10,11,12,13,14,15,  16,17-dodecahydro-1-Hcyclopenta[a]phenanthren-17-yl)  Hex-1-ynyl]-trime | 25.86 | 4.54 | C_30_H_48_O_2_Si |
| Astaxanthin | 26.10 | 3.30 | C_40_H_52_O_4_ |
| 4H-Cyclopropa[5',6']-benz[1',2':7,8]azuleno[5,6]oxiren-4-one,8,8abis( acetyloxy)-2a[(acetyloxy)methyl]-1,1a,  1b,1c,2a,3,3a,6a, 6b,7,8,8-adodecahydro6bhydroxy-3-amethoxy-1,1,5,7-tetramethy  l-,[1aR(  1aà,1bá,1cá,2aá,3aà,6aà,6bà,7à,8á,8aà) | 31.36 | 5.72 | C_27_H_36_O_10_ |
| Lup-20-(29)-en-3-one | 32.47 | 26.43 | C_30_H_48_O |
| 9,19-Cyclolanostan-3-ol,-24,24-epoxymethano,  Acetate | 34.00 | 30.56 | C_33_H_54_O_3_ |

**Supplementary Figure**

**Figure S1:** Dose and time dependent effect by AA extracts on viability of COLO-205 cells. (A) EAA and (B) AAA. Data presented as mean ± S.D (n=3) and compared as percent viability of control untreated cells vs. EAA or AAA treated cells. *p< 0.05 **p<0.01

**Figure S2:** AA treatment of COLO 205 cells leads to apoptotic changes as assessed by AO/EB staining. (A) Control, (C) EAA and (E) AAA observed for uptake of Acridine Orange dye and (B) Control, (D) EAA and (F) AAA observed for uptake of Ethidium Bromide (magnification 200X).

**Figure S3:** Evidence of apoptosis induction in AA treated COLO-205 cells by Annexin V/PI staining. (A) Control, (C) EAA and (E) AAA , observed for Annexin V staining and (B) Control, (D) EAA and (F) AAA for PI staining.

**Figure S4:**

β-glucosidase pre-treatment of AAA leads to enhanced cytotoxicity in treated COLO-205 cells. Data presented as mean ± S.D (n=3). A comparison was made between the treatments for each dose selected. *P<0.05, **P<0.01, ***P<0.001.

**Figure S5:**

Gas chromatography –Mass Spectrophotometric spectra of AAA extract.

**FIGURES**

**
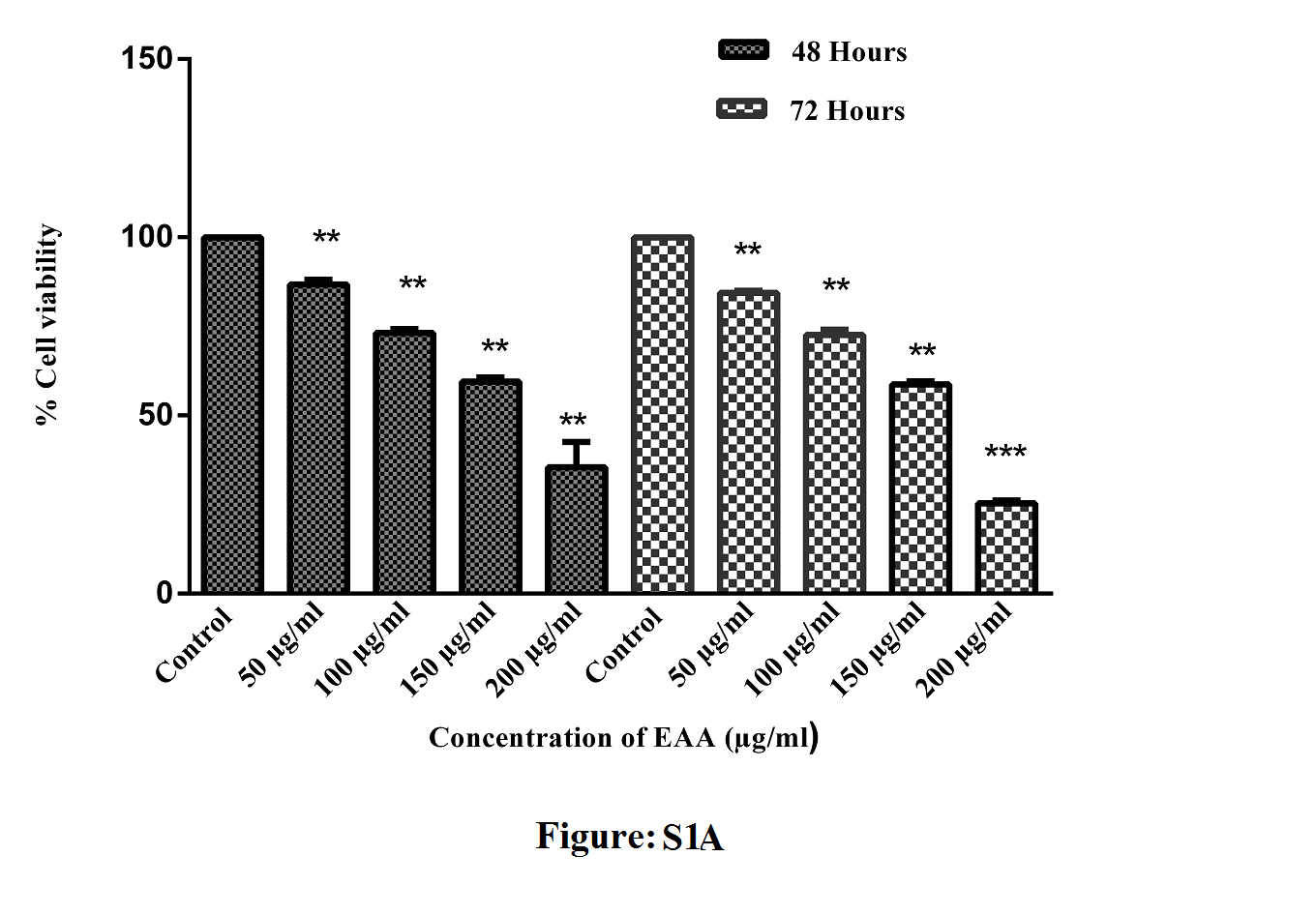
**

**
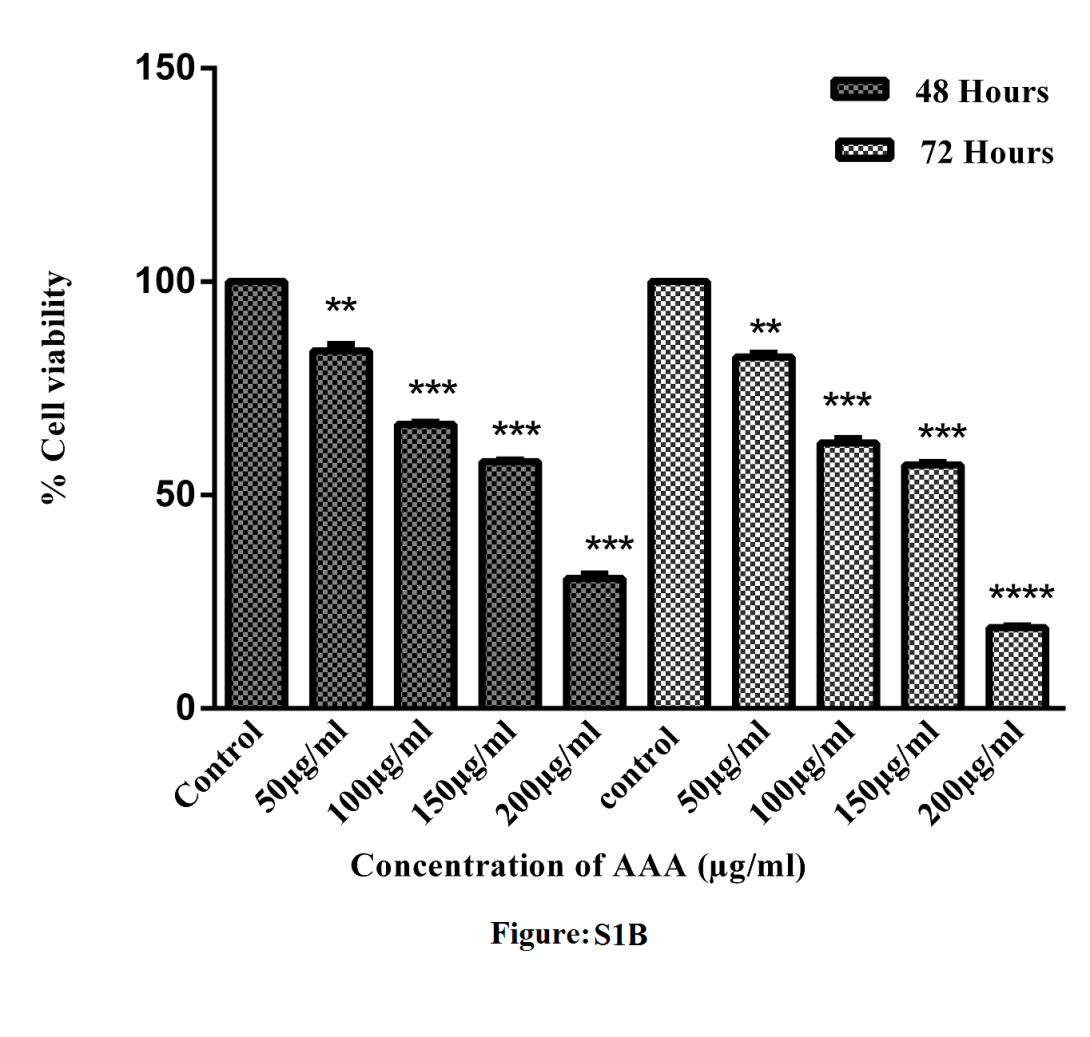
**

**
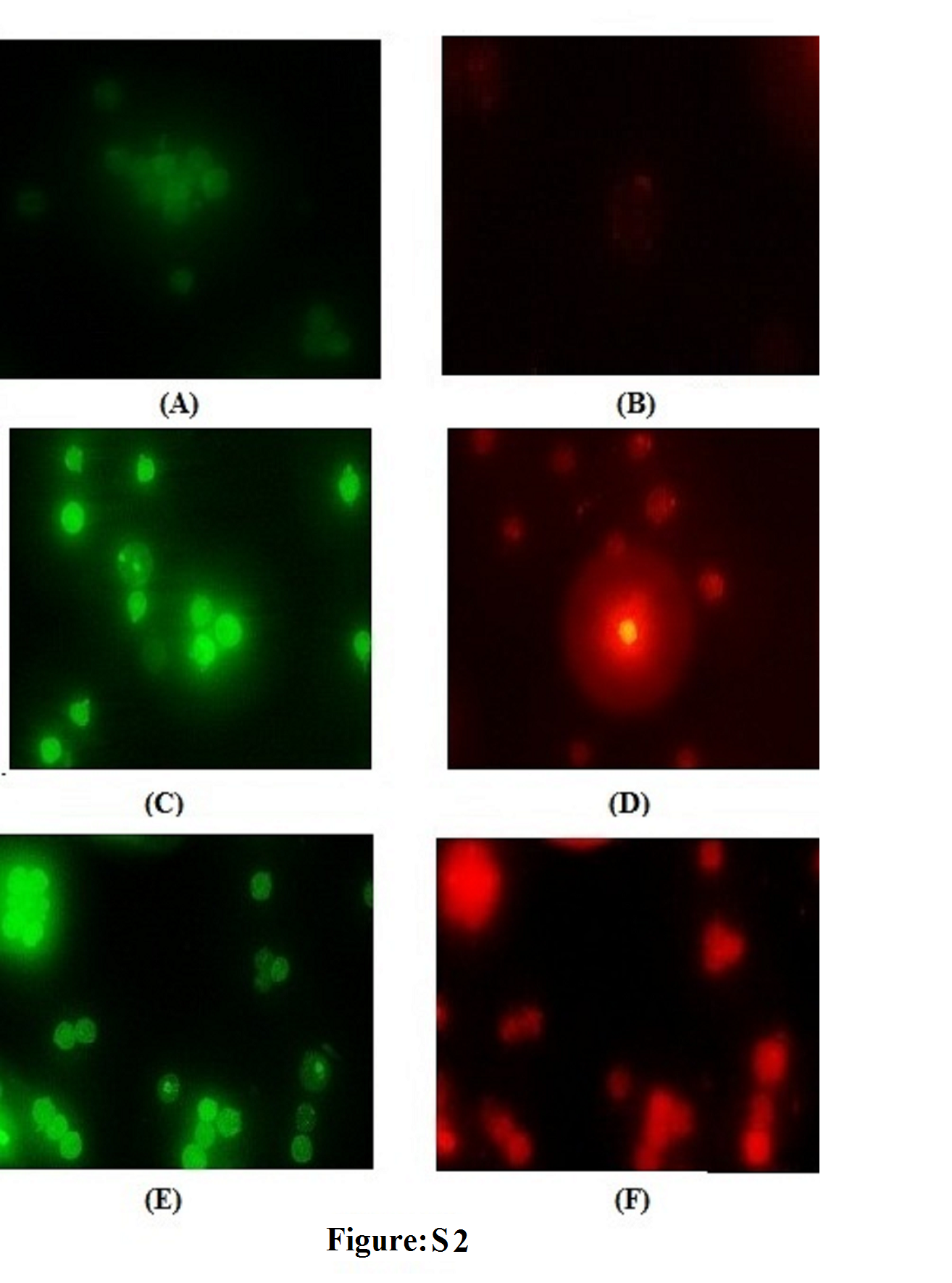
**

**
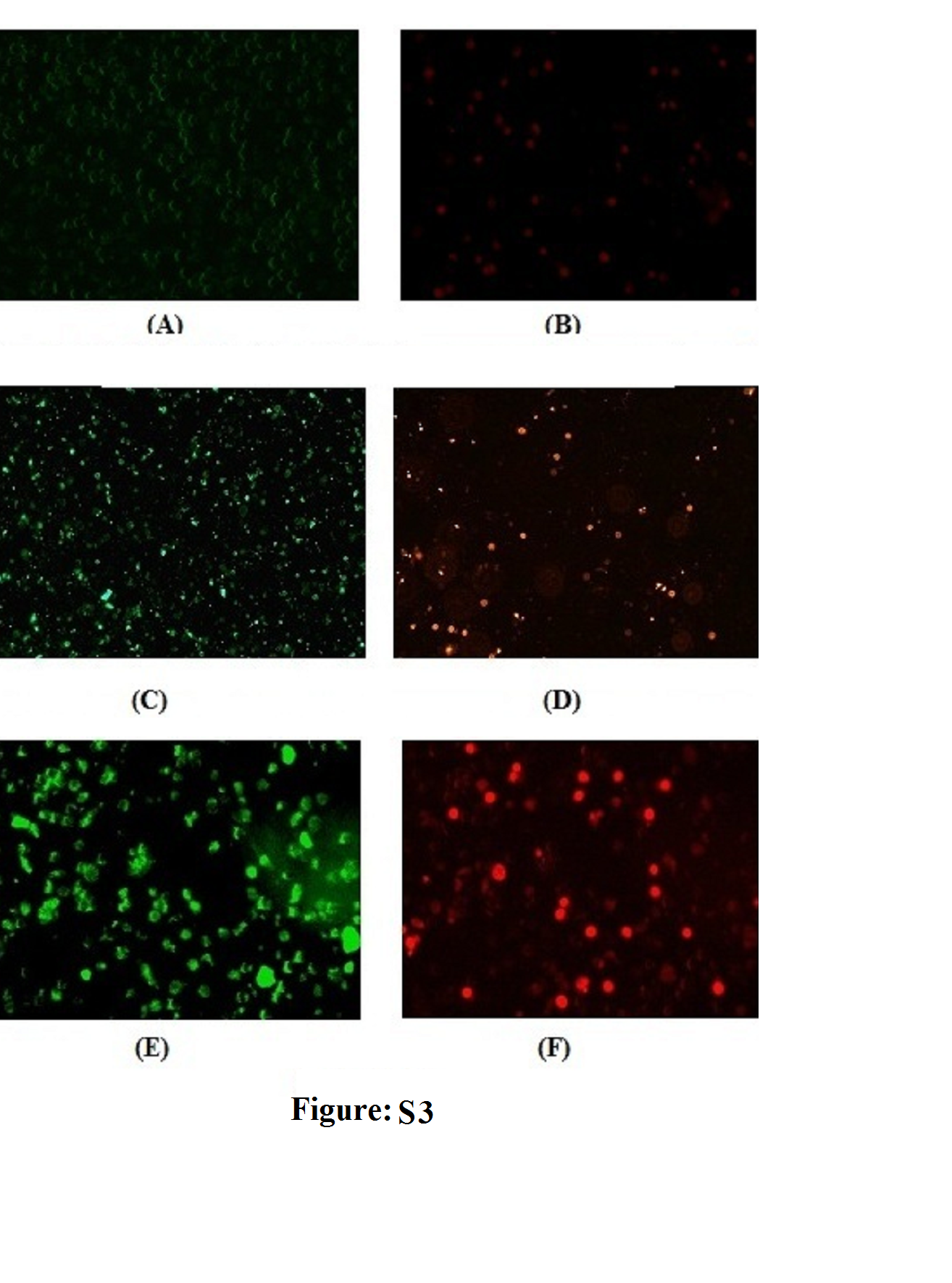
**

**
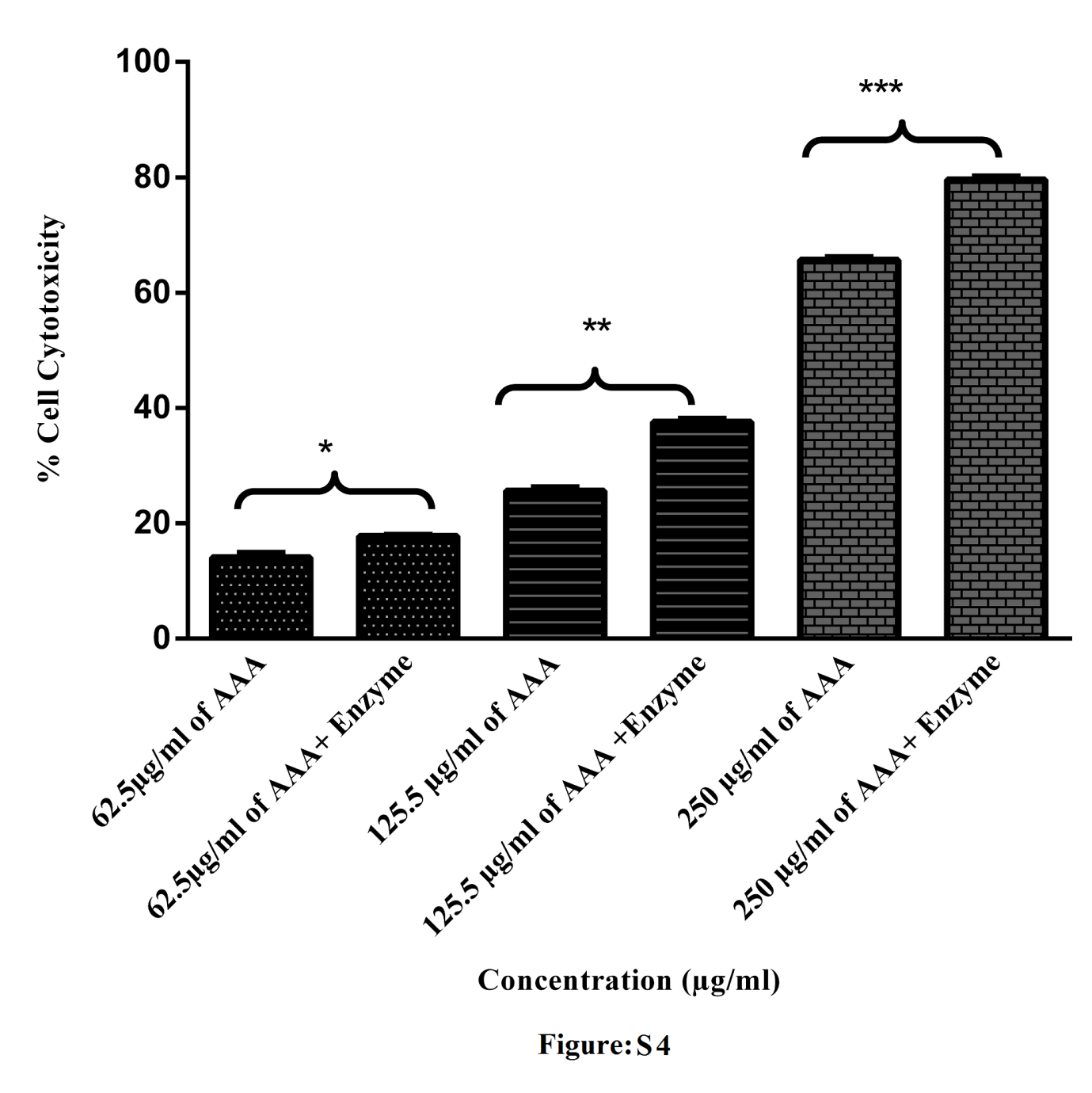
**

**
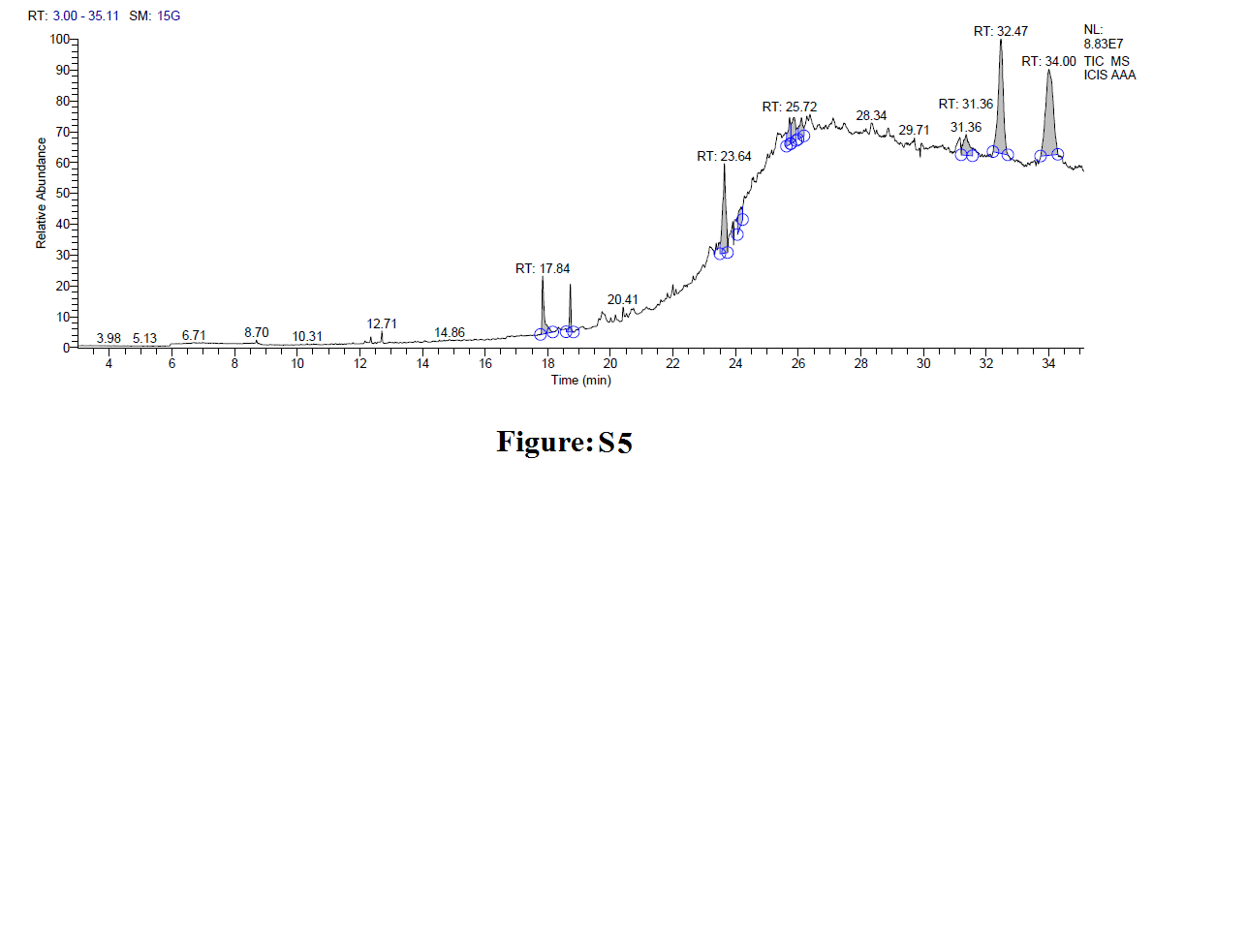
**
